# “The trip actually opened our eyes to things that we were supposed to do and we were not doing”: developing primary health care system leadership in a low-income country with peer exchanges

**DOI:** 10.1007/s43999-023-00030-w

**Published:** 2023-10-18

**Authors:** Mawuli Kushitor, Kalifa Wright, Adriana Biney, Edmund W. Kanmiki, Pearl Kyei, James F. Phillips, John Koku Awoonor-Williams, Ayaga A. Bawah

**Affiliations:** 1 The Department of Health Policy, Planning and Management (UHAS), School of Public Health (SPH), University of Health and Allied Sciences (UHAS), Ho, Ghana; 2 The Center for Health Information and Analysis, Boston, MA USA; 3 Regional Institute for Population Studies (RIPS), University of Ghana, Legon, Ghana; 4 Institute for Social Science Research, The University of Queensland, Indooroopilly, QLD 4068 Australia; 5 Heilbrunn Department of Population and Family Health, Mailman School of Public Health, Columbia University, New York, NY USA; 6 Policy Planning Monitoring and Evaluation Division, Ghana Health Service, Accra, Ghana

**Keywords:** Leadership, Leadership training, Health care system, Open system, Systems learning districts

## Abstract

**Background:**

Health care systems in low and middle-income countries are decentralizing and devolving power to the periphery. Transferring power without systematic processes to develop and nurture leaders at the district compromises the effectiveness and sustainability of the decentralized health system. To address this problem, we developed an approach to leadership learning by observation and experience that improved the organization and performance of the health care system in a district in Ghana.

**Methodology:**

Using two rounds of a longitudinal qualitative study, the study explores the determinants of implementing the Community-Based Health Planning and Services (CHPS) initiative in a district in Ghana. Insights were gained concerning the leadership regimes of two leaders who administered health services in a common geographic area at different points in time with remarkably contrasting outcomes. Insights of health workers who participated in both periods were elicited to clarify interview contexts. Ten focus group discussions (FGDs) and five expert interviews were conducted for each round of the study. The study was informed by a systems appraisal approach that utilized a thematic analytical framework.

**Results:**

Providing district leaders with a practical observational experience had a significant influence on health care delivery in all aspects of health care provision at the district level. Exposing participants to models of best practices facilitated the replication of processes that improved the conduct of service delivery and CHPS implementation. Upon reflection, district leaders attributed performance constraints to their lack of understanding of practical ways of responding to complex district health system development needs. Observation from community members, volunteers, and health workers who witnessed the system development period corroborated narratives that leaders had expressed.

**Conclusion:**

Effective leadership is optimally developed with participatory learning that provides leaders with direct access to fully functioning systems. Learning by observation can be structured and used to quicken the spread of managerial excellence.

**Supplementary Information:**

The online version contains supplementary material available at 10.1007/s43999-023-00030-w.

## Background

Over four decades, the Alma-Ata declaration has inspired hope across the globe for the implementation of health system leadership that would prioritize community participation over technical and bureaucratic health administration [1–3]. The promulgation of health care governance and practice that emphasize social justice, equity, bottom-up leadership processes, and grass-root participation acquired the overwhelming support of 134 governments and 67 international organizations [4]. As powerful as it was revolutionary, the Alma-Ata Accord lacked clear directions, policy guidelines, or strategies to comprise best practices for delivering *health for all* at the Primary Health Care (PHC) level. The current policy addresses this need by providing practical means of achieving the Alma Ata agenda as Universal Health Coverage (UHC). Yet, the progress of PHC development in developing countries has been constrained by overly centralized organizational systems [4–6]. Crucially, power transfer, management systems, health care governance processes, and structures to the periphery lack a solid evidence base. African countries, in particular, have sustained dysfunctional vestiges of colonial health care administrative structures over the years [7]. Colonial health systems were centralised in a few selected urban locations, primarily to cater for colonial workers and their dependants. Till date, most of the health care infrastructure in Ghana is concentrated in a few urban locations to the detriment of the vast majority of the rural populace. Compounding this problem, poorly thought through legislative instruments meant to decentralize systems have blurred the lines between vertical and horizontal programme goals [8–10].

Consequently, local system leadership capacity remains personality-dependent, rather than being grounded in systematic organizational processes. The pathway to *health for all* has been difficult and unclear. To address this gap in Ghana, a longitudinal qualitative study was conducted to provide informal leadership training to health care leaders in selected districts in the Volta, Oti and Northern Regions of Ghana. The Program for Strengthening the Implementation of the Community-based Health Planning and Services Initiative in Ghana (CHPS +) was a 5-year project launched in 2016 to strengthen the capacity of District Health Management Teams (DHMT) to oversee improvements in the delivery of primary health care. This was achieved by creating model facilities in selected districts. The model facilities created a continuous system of learning for appointed managers. This study therefore aimed to observe the influence of these leadership learning systems on the performance of the districts management leaders and their teams through an intervention known as CHPS + . For this study, we chose one district in the Volta Region of Ghana to fully observe the complex dynamics of leadership learning through the CHPS + programme. Details of the CHPS + project are published by Phillips et al. 2018.

Following the Alma-Ata declaration of 1978, the Republic of Ghana launched a primary health care policy like many countries in the developing world [11, 12]. However, evidence compiled in the late 1980s showed that this program was not achieving its “health for all” goals. Therefore, a phased program of implementation research was launched in 1994 to develop and test means of providing community-based primary health care [13, 14]. For 18 months, the Navrongo Health Research Centre in Northern Ghana conducted three community pilots to develop a community-based primary health care [12]. The outcome of this pilot was then tested as a district-level plausibility trial over the 1996 to 2003 period [13]. Early evidence of childhood survival impact led the Ministry of Health to adopt the Navrongo service model as national policy in 1999 through a program known as the Community-based Health Planning and Services (CHPS) initiative [15]. Although first implemented in 2000, a scale-up trial was initiated by transferring the Navrongo model to Nkwanta District of the Volta Region where replication was rolled out in ways that generated key lessons for guiding implementation of CHPS in other districts of Ghana [16]. The Nkwanta implementation process enabled Ghana’s operational policy planners to develop guidelines and milestones that have had important pragmatic effects on how CHPS should be implemented nationwide [17–19].

The core CHPS workforce comprises deployed community professional nurses, termed “Community Health Officers” (CHO). CHOs are responsible for primary healthcare service delivery that includes Integrated Management of Childhood Illnesses (IMCI), family planning, referral, and home visitation services. CHO and their supervisors commence implementation of the program by mapping a cluster of three to five adjacent communities where an operational “zone” is to be placed [20]. CHPS workers are trained to liaise their activities with the traditional system of governance. Outreach to community leaders is initially focused on convening a Community Health Management Team (CHMT). They are trained in CHPS oversight and tasked with the responsibility of recruiting Volunteer Health Workers (VHW). CHOs instruct the VHW to provide health education and promotion through community mobilization activities. The UHC is constituted from community volunteers and tasked to aid the administration and operation of CHPS and mobilize community members to attain communal health development goals. Together with the launching of a National Health Insurance Scheme (NHIS) in 2003, CHPS became Ghana’s flagship initiative for achieving Universal Health Coverage (UHC) [21]. In addition to the formal structure, the governance of CHPS involves marshalling traditional social networks for supporting supervision, who convene a Community Health Management Committee (CHMC). Nearly all towns and villages have a recognized conventional governance system comprised of chieftaincy arrangements supported by a panel of elders. While customs may vary somewhat by location and cultural setting, most local governance systems are well-understood by community members and sustained by social organizational norms that structure key relationships.

### The district health care system

The Ghana Health Service operates a three-tiered health care delivery system which is represented by CHPS zones at the periphery. CHPS staff report to supervisors based at Sub-District Health Centres (SDHC) that are staffed by a team of higher-level health care providers. Sub-districts, in turn, report to District Health Management Teams (DHMT) comprised of paramedics who coordinate various health service functions at the level of the entire district. Each DHMT is headed by a District Director of Health Services (DDHS). District hospitals and DHMT report to regional tertiary care hospitals located in each regional capital. This study focuses on the operations of CHPS compounds. CHPS compounds are the lowest care facilities located in rural communities. CHPS is Ghana’s main means of providing UHC to peripheral communities. Even though CHPS compounds are an integral part of the district health system, they are often neglected, under resourced and poorly managed. Unlike most of the health care system the operations of CHPS is different in its operations, in that it is co-managed and co-sponsored by the local community. This requires a thorough understanding of the formal health care system governed by biomedical scientific knowledge and also an understanding of the social system of communities to build the CHPS compounds.

Despite evidence that CHPS saves lives and contributes to reproductive health when its services are fully functioning, several studies show that the pace of CHPS scale-up has been unacceptably slow and that implementers have often failed to replicate services and strategies that contributed to the Navrongo experiment’s success [22, 23]. Key amongst the challenges reported is the recurrent failure of CHPS implementers to involve local communities effectively and to sustain the involvement of Volunteer Health Workers (VHW) who were an essential component of the Navrongo model [23]. While several studies have identified CHPS implementation lapses, few have provided evidence-based solutions for addressing these challenges. The CHPS + project, a partnership between Mailman School of Public Health in the United States, The Regional Institute for Population Studies (RIPS), University of Ghana, The University of Health and Allied Sciences (UHAS), Ho in Ghana and the University for Development Studies (UDS) also in Ghana, is implemented to improve the operational efficiency of CHPS in Ghana. The CHPS + project study aims at clarifying the means for reviving the potential for CHPS to offer a nationwide pillar for attaining UHC.

We compile evidence from a district where the pace of CHPS expansion was initially unusually rapid, resulting in improvements in health indicators reported over four years. Based on established district performance criteria for health system coverage and care, the study district was adjudged the second-best performing district for primary health care services in a region of Ghana. However, when the District Director of Health Services (DDHS) was transferred and replaced, performance during the operational leadership era of the replaced DDHS was associated with a marked performance deterioration. For this paper, we designate the former director as “DDHS-1” and the current director as “DDHS-2.” We utilize this operational variance to assess stakeholders' perspectives of the two contrasting leadership regimes by conducting open interviews of health workers and community leaders responsible for primary health care functions during both DDHS leadership eras. While the challenges to the scale-up of CHPS are well-diagnosed, little is known about feasible means of addressing CHPS leadership lapses [24–27]. In particular, research is yet to compile and interpret observations that are based on the reflections of successive managers and support staff concerning ways to improve CHPS leadership. To address this knowledge gap, we compile the observations and recommendations of the health workers who experienced these contrasting managerial styles. We premise our investigation on the comparative analysis of before versus after the leadership change. Quite interestingly, the stark contrasts in personal reflections of DDHS-2 before CHPS + and afterwards shows leadership training is crucial to the governance of the district health care systems. Participatory evaluation is employed to facilitate the appraisal of effective leadership for primary health care delivery. Open systems research focuses on the complex relationships that define work routines, power dynamics and reciprocity, and the flow of information and knowledge between systems [28, 29].

## Methods

### Conceptual framework

#### CHPS as an open system

A system is defined as a group of interdependent components that interact together to form a complex and a unified whole that achieves a goal. All systems have *elements*, *interconnections,* and a *function or a purpose*. A system survives by the multiple interactions between and within the different components. An open system is defined by the broader context in which a system functions [30]. In community health services, the social system defines program success by jointly influencing health-seeking behaviours in individuals and families through the participation and support of traditional leaders of community governance. CHPS thus operates as a complex open system because it is embedded in both the bureaucratic layers of the Ghana Health Service formal organization and the traditional social system that structures community organization. To function effectively, these two systems must work in concert to produce optimal functioning of service delivery. Interconnections shape the flow of information between the system components, the resilience of programme governance, and the provision of essential materials and human resources. Interconnections also define norms that govern organizational behaviour, both within the health system and social system. For open systems, such interconnections thus bring life to a system. Although CHPS has several elements, these defining interrelationships govern the delivery of service in ways that actually produce the health outcome. Open systems thinking is a field that has been embraced by several theoretical and programmatic planning approaches [31–33].

### Study design

This study adopts Participatory Action Research (PAR) design where research subjects are effective contributors to the study of their own reality. This concept is based on the premise that research subjects are more likely to take the necessary action to advance their own lives when they are part of the process of knowledge production. This process allows research participants to become more reflexive of their environment, enhancing their level of awareness [34]. The awareness created from the participants empowers subjects to take action for themselves. PAR comes in various forms and has been used to study and empower local communities to own their livelihoods and take charge of it [35–37]. PAR is known to release the *spirit of inquiry* into research subjects where they are active consumers of research involving their environment and, more importantly, leading them to ask relevant questions for themselves. This CHPS + study design allowed researchers from four universities and actors within the health system at the national, regional, and district levels and community members to form research/learning alliance. The knowledge acquired is directly utilized to improve the delivery of health care within rural communities. This collaboration is timely as it comes when the national scale-up of CHPS is running into serious challenges, particularly with significant deviations from the original Navrongo experiment's original tenet [23, 38]. For example, there is overwhelming evidence to show that volunteerism which is core to the implementation of CHPS has significantly atrophied over the years with the national scale-up [26, 39–41].

### Data collection

This study was carried out in a district in Ghana. Data collection was carried out in April, 2017 and February, 2019. The study applied a mix of qualitative methods, including Focus Group Discussions (FGDs), expert interviews and structured observational technics. FGDs were conducted with frontline health workers (this comprises community health nurses, enrolled nurses, midwives, and depending on whether they received extra training they may be deemed as CHOs), Community Members and Volunteer Health Workers. In total, 10 FGDs and five expert interviews were conducted. These interviews were conducted in both the first and the second rounds of the study. More importantly, views of the district director at the time when CHPS was initiated and those of the current district director were systematically analyzed. For the current director, two periods were recognized. The period before the CHPS + project implementation and during the period of implementation. These distinct phases for the current director make provision for how the learning experience of the project affected healthcare administration within the district. The FGDs were conducted with volunteer leaders in the district who shared their common experiences of neglect and isolation. FGDS were also conducted with sub-district leaders, staff of the DHMTs at the district offices. These shared experiences of CHPS from different cadres of health providers associated with CHPS helped to improve the understanding of the system from multiple perspectives. Individual interviews were conducted with the director and their assistant before and after the CHPS + intervention study.

Participants were purposely chosen based on their professional roles and duration of stay in the study communities. The District Health Directorate hosts the DHMT while a community hosts the CHPS zone. We interviewed every available health worker associated with a CHPS facility in selected CHPS zones in this particular district. Summaries of the interviews conducted are contained in Tables 1 and 2.

**Table 1:** Participants by respondent category

FGD Participants in the district				
	Area 1	Area 2	Area 3	Total
DHMT	4	0	0	4
CHOs	7	0	0	7
Volunteers	0	7	4	11
Leaders	0	5	11	16
Total	11	12	15	38

Source: CHPS + study baseline and midline,2017 and 2019

**Table 2:** Specific characteristics of respondents at the DHMT

Characteristics	Central Tongu
**Position/Role in DHMT**	
District Health Director	^+^
District Administrator/Senior Executive Officer	^+^
District Public Health Nurse	
District CHPS Coordinator	^+^
District Health Promotion Officer	^+^
District Nutrition Officer	
District Health Information Officer	
District Disease Control Officer	^+^
District Storekeeper	
District Accountant/Senior Finance Officer	
Senior Midwifery Officer	
District Claims Officer	
Community Health Nurse/Officer	^+^
**Sex (%)**	
Male	75.0
Female	25.0

Source: CHPS + study baseline (2017) and midline (2019

^*^The CHPS Coordinator plays also doubles as the Disease Control officer in the district

^+^indicates that the health care professional was interviewed(Not all districts have the full compliments of health staff, those that were present in this district were identified)

Within the DHMT, any professional whose portfolio affected the CHPS process was recruited for the study.

Both the individual interviews and FGDs lasted an average of 60 min. Two FGDs went beyond 90 min. The interviews were conducted by the research team including faculty members from the University of Ghana. Some trained interviewers were recruited to be part of the research team. The training lasted for three days before the fieldwork. For consistency with data collection, the same teams were maintained for all the rounds of the study. During the training, the research team role- played the interview guides both in English and in the local languages. This was to ensure consistency of administering the guides.

### Ethical considerations

The study protocol was reviewed by the Ghana Health Service Ethical Review Committee, Accra, and by the Columbia University Institutional Review Board in January 2017. Written informed consent was obtained from all literate study participants. Participants who illiterate were asked to thumbprint the consent forms to indicate consent. Refreshments and soap were provided to all study participants as reciprocity.

## Analysis

All interviews were recorded via the means of audiotapes with the expressed permission of the research participants. Verbatim transcriptions were carried out by trained transcribers with competencies in the local language. The transcripts were quality checked by an independent person with both language and grammar competences to ensure that the content of the transcripts were accurate and also readable.

For this study, a code was defined as a basic unit of an idea. The coding process involved the identification and labelling of ideas contained in each transcript. This was after an exhaustive period of extensive reading of the transcripts by each analytical team member to familiarize themselves with the texts. The coding process was informed by two primary considerations, deductive and inductive. The deductive coding involved identifying and labelling pre-existing ideas on CHPS and primary health care in Ghana [27, 38, 42–44], while inductive coding referred to discoveries emanating from the transcripts. We found that some of the leadership engagement text were coded as inductive codes, which means some of the ideas expressed were novel. The coding was done by a team of six, four from the School of Public Health (SPH), University of Health and Allied Sciences (UHAS), and two from the Regional Institute for Population Studies (RIPS), University of Ghana. Prior to the coding process, the team had three separate meetings to agree on the constitution of a code and labelling. This was to ensure similar ideas occurring in any transcript analyzed by team members would be labelled or coded the same way in Atlas.ti. After the first round of coding, through *constant comparison* where observations from all transcripts were compared, a table was generated in excel to align all the ideas in all transcripts against each other. From this table, dominant ideas and less dominant ideas were observed as well as the saturation point. This table was generated for both the baseline and the midline qualitative rounds of data collection. This helped the research team quickly identify the clustering of ideas and themes within each transcript and across the entire data corpus. The last part of the analytical process was grouping codes associated with a particular idea. For instance, all codes associated with volunteers were grouped under one thematic area. For the two directors, each theme or idea expressed, the perspectives of the former and current directors, and other health workers were observed. This allowed the research team to understand the processes that were adopted, the reasons for their adoption and the consequences that followed.

The study adopted a participatory research approach where research participants were involved in the design and implementation of the study from the inception to its conclusion.

## Results

### Observations in the field

The study team arrived at the district and immediately proceeded to visit a nearby CHPS zone. The CHPS health post was deserted, and generally unkempt. For three months, electrical power had been disrupted, depriving CHPS of the capability to store medical supplies. The CHO in charge explained that community leaders had refused to pay for electricity in running the compound because of an unresolved conflict with the district political administration concerning resources for refurbishing the local market. The team engaged in subsequent inquiries involving traditional and district authorities. The observations from these interviews provided insights into how CHPS operated in the community. During the observational tour, the study team learned that volunteerism had atrophied throughout the district. This was confirmed by the current district director, who noted that only 5 of the 120 volunteers who were trained were still active. Clearly, the collapse of volunteerism was not an isolated event but was symptomatic of a sustainability failure. There was a general consensus amongst the various stakeholders in the district that CHPS had lost much of its original service capability. Major components of the CHPS process were not functioning. As the DDHS noted:

*Quote 1 and 2* in Additional file 1.

Volunteers also expressed this view. For example, a VHW noted:

*Quote 3* in Additional file 1.

The initial interviews and field encounters yielded four observations of system disconnectedness: i) there was a marked disconnect between CHO task leadership at the CHPS facility level and health care leadership at the district level, ii) there was a disconnect between CHO leadership and the community volunteers, iii) the health system leadership virtually ignored interaction with the traditional and municipal authorities, and iv) the quality and content of CHPS services malfunctioned from the sub-district level down to volunteers and the community, largely because of lapses in support for utilities and essential supplies.

While volunteerism had become marginal to CHPS in the study district, this was not the case in the past. Volunteers noted that the previous DDHS leadership encouraged all supervisors and CHOs to implement volunteerism, a point of administrative and communication focus that the current DDHS neglected. A volunteer described this climate of transition from vibrant management to malaise:

*Quote 4* in Additional file 1.

Volunteers were clear about DDHS commitment to facilitate their participation in ways that ensured that VHW task and responsibilities were respected and impactful. VHW attributed the success of their involvement to DDHS leadership at the time. The successful district leader attributed program success to activities that he could implement and control:

*Quote 5* in Additional file 1.

However, the successor DDHS attributed problems with volunteerism to circumstances beyond the control of the DDHS, such as remuneration policy:

*Quote 6* in Additional file 1.

These statements attest to the importance of direct involvement and communication. DDHS-1 engaged the volunteers directly, the DDHS-2 attempted to engage the volunteers through the CHMT. However, the CHMT was also neglected and had become inactive. The structure of system support for volunteers had almost collapsed, leaving them to feel neglected and not needed. For two years, the DDHS-2 expected the community health management team to oversee and resource the volunteers without direct involvement. Unknown to DDHS-2 however, the community management committee had long collapsed with the decreasing participation of the volunteers. The low involvement was therefore not an isolated event but a process that gradually failed evidenced by the gradually low participation of the volunteers. Further, because DDHS-2 was not directly involved, DDHS-2 did not notice the gradual, systematic failure.

**Fig. 1:**
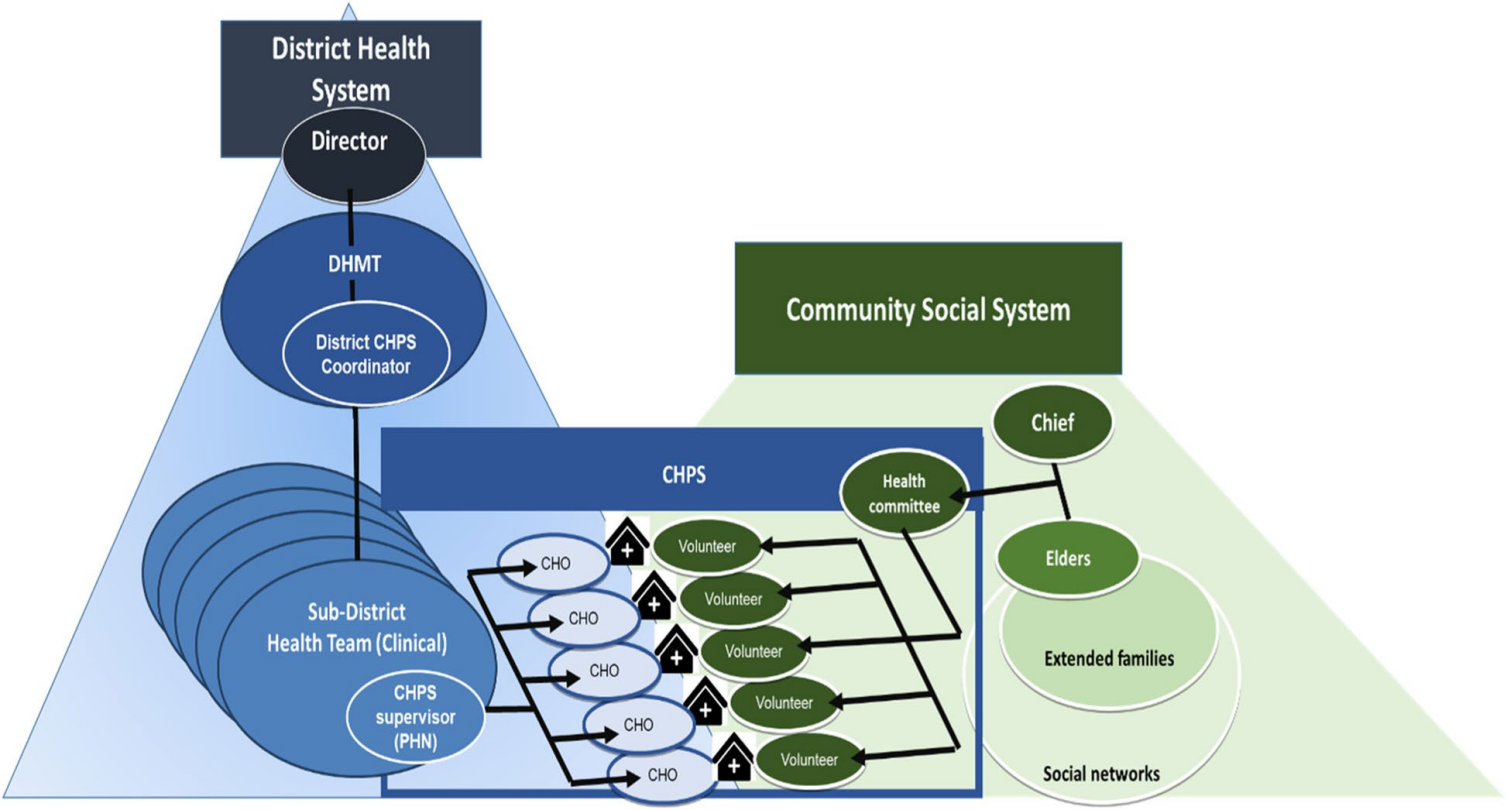
CHPS as an open system

Based on our field observations and the information from the conceptual framework, CHPS is appropriately designed in Fig. 1 to portray all the relevant components and the interactions within them. Figure 1 portrays the open systems concept of leadership, in which linkages within the health bureaucracy link with community leadership. The blue section represents the formal system while the green represents the informal administrative structure. The black figure with the cross is the CHPS facility. The position of CHPS at the interface of these structures demonstrates the role of its mechanisms in linking the two systems, if CHPS is functioning optimally.

In summary, CHPS is nested within the Ghana Health System structure, and draws upon support from the community social structure. Understanding CHPS requires a framework that one pays attention to the connection between the different actors within an evolving social system.

#### Learning leadership through lessons from Navrongo

Although, discussions appeared to show that DDHS-1 had better outcomes from the CHPS process, the information below reveals that DDHS-1 was supported from the regional level, something DDHS-2 did not have prior to CHPS + implementation.

### Quote 7

More recently, activities of a CHPS development project enabled the study DHMT to visit the Upper East Region as well. This exchange had immediate implementation effects:

### Quote 8

The discussions on the Navrongo experiment from both DDHS-1 and DDHS-2 reveals the impact of the Navrongo experimentation on the implementation of CHPS. The climate of health policies and leadership at the time favoured the initiation and the implementation of CHPS. The wealth of knowledge and experience gained by the previous directors made important differences in the administration of CHPS. Especially, the community involvement and the deployment of VHW. Unfortunately, these useful lessons were not passed on systematically to successive directors.

*Quote 9* in Additional file 1.

The attitude of directors with Navrongo experience towards volunteers is very different from those who did not have that experience, a finding that has been noted in reference to lack of fidelity to CHPS milestones in general [44]. The absence of continuous participatory learning within the study district had significant ramifications. The decline of the Navrongo-Nkwanta process could have been averted with the introduction of effective participatory knowledge management. This mode of leadership education merits attention in the planning of leadership training for CHPS initiation, implementation, and routine management. The absence of key leadership learning was noted with respect to the collapse of the collaborative process between the various stakeholders and the community. In particular, there was limited focus on finding resources to enhance the commitment of community volunteers to provide services. Training for leaders and volunteers was neglected, and supervisory support for volunteer activities declined. Consequently, the sub-district health system lapsed into a passive clinical program that neglected the elements of community engagement that were critical to catalyzing volunteerism and for the overall implementation of CHPS resourcing and functioning.

Collaborative process between the various stakeholders is simpler to demonstrate than to document as a training syllabus. Community leaders typically welcome engagement processes. As the directors noted:

*Quote 9 and 10* in Additional file 1.

These statements illustrate the contrasting approaches to community engagement of DDHS-1 and DDHS-2. The former DDHS was emphatic about the importance of involving local and community collaborators. Specifically, these collaborators were community members and members of the community governance system. But DDHS-2 was apparently unaware of the community engagement processes that had successfully gained support for CHPS among local and district authorities. DDHS-2 had not benefitted from the strategies that were instituted by his predecessor. This lapse in knowledge management needlessly deprived CHPS of volunteer human resources for CHPS facility construction that had worked so well in the past. The DDHS-1 was proactive in looking for ways to accommodate CHPS operations in interim facilities so that services could start without delay.

*Quote 11* in Additional file 1.

But for DDHS-2, implementation challenges were perceived to be someone else’s problem:

*Quote 12* in Additional file 1.

Resource mobilisation is the most challenging aspect of the CHPS process. In principle, both the GHS national and the local community are expected to provide inputs to sustain the activities of CHPS. The GHS provides staff, supervision, medication and other medical supplies, while communities are expected to provide CHPS with basic accommodation for staff, security for CHO, maintain the health post premises and seek ways to support the volunteers. Collaboration is thus critical to the optimal functioning of CHPS. But, in the absence of strategic engagement from the DDHS-2, the different partners grew apart, resources dwindled until there was nothing left to run CHPS and the once optimal functioning of CHPS disappeared.

*Quote 13, 14, 15* in Additional file 1.

The quote above illustrate the multi-dimensionality of partnership arrangements that are critical to overcoming resource scarcity. Fundamentally, the absence of effective volunteer engagement is symptomatic of neglect of community members in general. As community involvement waned, political accountability for sustaining essential public infrastructure also atrophied. CHPS lost key resources: volunteer labour for CHPS facility construction, volunteer security guards for health posts, and public revenue for electrification of CHPS facilities. Even though funds were available, system dysfunctions deprived CHPS of critically needed support. As DDHS-1 noted, his support was crucial to CHPS functionality and sustaining this support was manageable:

*Quote 16* in Additional file 1.

But, the DDHS-2 viewed support lapses as disruptions that were external to district team intervention and control. These were described passively as problems, without mention of proactive steps that could address such problems. For example:

*Quote 17* in Additional file 1.

Beyond equipment, there were systems designed to remunerate and acknowledge volunteer contributions such as i) involving volunteers in mass immunization campaigns activities where they would get modest compensation, ii) involving volunteers in direct provision of essential services to community members for a token fee or iii) community contributions in cash or/and in kind as an expression of appreciation for their contribution to community health:

*Quote 18* in Additional file 1.

However, the current DDHS tended to emphasize aspects of CHPS services that VHWs were not intended to pursue. Discussion often focused on the negative:

*Quote 19* in Additional file 1.

Although volunteers are expected to provide their services willingly without demanding payment, the two DDHS approached this sensitive topic differently. The DDHS-1 attempted to find revenue through whatever means could be pursued’ whereas the DDHS-2 felt that VHW should not expect to be paid. If payment was required, it was the responsibility of the CHMC to find resources for the volunteers. DDHS exercised their discretion over the decision to pay or not and even payment approaches. Obviously, volunteers preferred to be compensated and were accustomed to receiving some income. NGOs compromised official policy by paying volunteers for their services, thereby arousing expectations that the government would embrace this policy.

The DDHS-2 worked in the district for a number of years before serving as a director. Yet, her experience lacked adequate exposure to CHPS operations and pragmatic ground-level leadership. A recent team exposure to the essential elements of CHPS operations was arranged as a tour of GEHIP operations in the Upper East Region. This exchange was useful because it provided DDHS-2 with an opportunity to reflect on ways that leadership could have been more effective. Crucially, once DDHS-2 could interact with Navrongo counterparts, the implementation process was clarified. Three implementation themes were particularly impactful, most importantly practical insights into community mobilization and volunteerism:

*Quote 20* in Additional file 1.

Learning about the implementation process that DDHS-2 alludes to was facilitated by direct observation of the Navrongo operation. All key managers teamed up with counterparts in Navrongo who could address questions and demonstrate solutions to problems.

*Quote 21* in Additional file 1.

Yet, direct support from the Regional Health Administration was also critical. As DDHS-1 noted: The DDHS-1 received financial support for CHPS implementation from the regional health administration at the time. Moreover, DDHS-1 benefitted directly from implementation learning conveyed by the Navrongo exchange. DDHS-1attracted resources from both the traditional authorities and the district authorities. And, to sustain operations, DDHS-1 engaged the volunteers personally.

Although DDHS-1 was highly successful with CHPS implementation, challenges were a continuous source of concern and action:

*Quote 22* in Additional file 1.

While direct DDHS-1 action built CHPS, failure to adequately delegate authority to DHMT deprived CHPS of functionality and sustainability. While DDHS-1 personally benefitted from the exchange with Navrongo, leadership learning acquired did not translate into concrete steps to sustain CHPS. The leadership capabilities of the DDHS-2 were enhanced by exposure and learning from the exchange with the GEHIP team in the Upper East Region:

*Quote 23* in Additional file 1.

The learning resulted in two significant systemic responses to the health systems. These were the revamping of the sub-district and CHMC in all CHPS zones. These two mid-stream management structures had important and immediate consequences for the community and its volunteers. The sub-district health system was restored to proper functioning. The quotes below and others reveal important changes that had taken effect.

*Quote 24* in Additional file 1.

Another significant effect of the leadership learning experience was revamping the CHMTs in order to improve oversight of volunteers.

*Quote 25* in Additional file 1.

There was a strong emphasis on direct community engagement in all interviews and discussions. For example:

*Quote 26* in Additional file 1.

In response to the exchange, the DDHS-2 placed a strong emphasis on training at all levels.

*Quote 27* in Additional file 1.

This capacity-building commitment was associated with renewing the climate of supervisory support, as noted by DDHS-2 in reference to recent changes:

*Quote 28* in Additional file 1.

A structured emergency response system has also been developed, with the acquisition of motor tricycles for the district coupled with motorbikes and mobile phones:

*Quote 29* in Additional file 1.

## Discussion

This study applied a longitudinal mixed qualitative study. Specifically, this consisted of FGDs, expert interviews, and structured observational techniques to explore the management and the delivery of health care within the context of a district. Leaders reflected on their current and past performances, coupled with shared experiences from several health workers who lived through the period of both leaders, provided insights into governing the health care system within a district. Key aspects of the leadership involved 1. Recognising the complexity of the district health system 2. Finding needed resources 3. The skill of combining the formally trained health personnel with community folks who are governed by tradition. 4. Getting the buy-in of the community to support and sustain CHPS and 5. Logistics and pharmaceuticals. Informed and skilled leadership was required to organize all this into an effective health system that actually met the constituents' needs. Observations from the study revealed that learning-*by-doing* was an effective way of teaching leaders to govern and lead effectively.

Contrary to this observation, the Ghana Health Service (GHS) provided intermittent refresher training for district managers. Most of these training sessions have been based on didactic classroom-style teaching. These episodes of training are often conducted in hotels that are isolated from the districts where participants are assigned. While these secluded training sessions have the utility convening district managers together to learn from one another, the direct contribution of these leadership training to actual management practices limited. Several studies of health systems in LMICS have concluded that leadership is defective [45, 46]. Unfortunately, the mode of leadership training that is prescribed typically focuses on capacity building without actual observational experience. The specific knowledge that spans the complex domains of personnel management, community relations, health technology, and resourse management spans the routine functions of an effective district manager. Leadership of a health system is far more complex and requires more than is possible to capture in a few days of classroom-style lectures [10, 47]. Perhaps, the preponderance of evidence on poor district leadership and management may reflect the ineffectiveness of these didactic training sessions [48–50]. Observations from this study revealed that district leaders are often assigned to this position with little practical training and preparation. Although there is evidence to show that most district managers have attained some management training certification in centres of higher learning, appropriate models of specific *know-how* may be practically beneficial in governing the districts effectively [51].

Observations from this study rather reveal that effective leadership is a learning experience. *Learning by doing* or *learning by observation* based on practical observations gives leaders a *hands-on* experience of leading a district. Leadership by learning and observation is not a new concept, this idea has been supported by over 40 years of social psychological research [30]. Based on this principle, Systems Learning Districts (SLDs) were created by the CHPS + projects to bring this experiential learning closer to district managers in four regions. Creating these miniaturized centres of learning in the regions is the first step in mainstreaming the provision of practical experiential training to leaders in a systematic way. Although this study is not a quantitative study that may allow for generalization, the systematic processes associated with decentralization are common phenomena to most health care systems in many developing contexts. While a direct application may not have been tested, the core ideas and principles are relevant for these jurisdictions as many health systems have followed a path of decentralization after the Alma-Ata declaration.

This general observation has potentially important policy implications, not only for the management of leadership training, but also for the management of the utilization of implementation innovation. Ghana has a three decade history of establishing rural research stations for testing means of improving health system functioning [52]. And, this legacy has been associated with important innovations, such as the development of CHPS as well as trial of its scale-up through replication research [53, 54]. Yet, the development of leadership training has yet to institutionalize the concept of team learning [55] or to embrace the observational model as a paradigm for improving health system functioning. Achieving this goal would involve creating learning districts in each region where leadership training can be conducted in concert with system observation and pragmatic learning.

### Limitations

This study was based in a single district of a region in Ghana. As such, unique characteristics of the region and locality may have affected results in ways that compromise generalization. Caution is warranted in the course of deliberations on national policy implications of results. Yet, the consistency of participant commentary suggests that participatory observation methods employed as an intervention merits systematic policy review and replication elsewhere in Ghana. The GHS has employed didactic classroom leadership workshops throughout Ghana without an equivalent imposition of qualitative systems appraisal. Results of our investigation attest to the need for systematic review of leadership development strategies, and consideration of replicating the CHPS + demonstration and exchange approach at scale.

## Conclusion

CHPS is built on relationships that define the bridge between the health system and the social system. A manager’s ability to nurture productive relationships involving various stakeholders profoundly influenced resource mobilization for the provision of primary health care. A leader must direct attention to both formal and informal administrative structures that may directly or indirectly influence the design, set up and regular functioning of CHPS.

Leadership learning is fundamental to the CHPS implementation process. To achieve a sustainable and effective implementation of CHPS within the context of the national scale-up, leaders require direct observational learning experiences that are planned, systematic, and conducted at regular intervals. CHPS coverage has been expanded by the training and deployment of community workers to the extent that it is approaching the national achievement of UHC at long last. Optimal functioning of the extensive national investment in CHPS manpower, equipment, and facilities requires grassroots implementation leadership that is often lacking. Developing this critical leadership capability through experiential learning will be crucial to the effective attainment of UHC in Ghana in the future.

### Supplementary Information

**Additional file 1.**

## Data Availability

Data will be made available upon request. Data can be requested from Prof. Ayaga Bawah of the Regional Institute for Population Studies. His email address is: aabawah@gmail.com.

## Abbreviations

ANC: Ante natal Care
CHC: Community Health Compounds
CHO: Community Health Officers
CHMT: Community Health Management Team
CHPS: Community-Based Health Planning and Services
DDHS: District Director of Health Services
DHMT: District Health Management Team
FGD: Focus Group Discussions
GHS: Ghana Health Service
MDGs: Millennium Development Goals
PPME: Policy Planning Monitoring and Evaluation Division
PAR: Participatory Action Research
SDGs: Sustainable Development Goals
VHW: Volunteer Health Workers
NGOs: Non-Governmental Organisation
GEHIP: Ghana Essential Health Interventions Programme
LMICs: Low and Middle Income Countries (LMICs)
SLDs: Systems Learning Districts
