# Supplementary material for: “The trip actually opened our eyes to things that we were supposed to do and we were not doing”: developing primary health care system leadership in a low-income country with peer exchanges
Source: Res Health Serv Reg. 2023 Oct 18;2:15. doi: 10.1007/s43999-023-00030-w (PMC11281752; doi:10.1007/s43999-023-00030-w)
Supplement: Supplementary file 1 — Additional file 1. [file 43999_2023_30_MOESM1_ESM.docx]

|  | **Quotes** |
| --- | --- |
| 1 | *“The volunteers were so important before we could regard the zone as completed. Without them, no completed zone. Over the years, CHPS turned into mini-clinics, with the loss of household service delivery components.”* (DDHS-1) |
| 2 | *“But for some of them, the spirit of volunteerism is going down because they have been working for free for a long time.”* (DDHS-2) |
| 3 | *“When they first started, we were 16 volunteers, and we were divided into groups. Some were trained on TBA, others on how to attend to malaria patients. These people have all stopped; when they call them for meetings they don’t come. We also have expectations, so we will not choose to stay if nothing is given to motivate us.” (*Community Health Worker FGD) |
| 4 | *“The first director to train us was outstanding. He gathers us and occasionally makes us feel good. We eat and drink, share ideas and our problems on the ground, but nothing of that sort happened when he left. That was in 2009. The current one does not even know some of the volunteers. We are the people working on the ground for the director's work to be easier but here is the case where he does not even know us, much less to appreciate us. This place was the first community that the CHPS was built so many people from other villages come to do a lot of workshops and training for their workers. We train some of the nurses on how to weigh the children, but now we are feeling a bit reluctant because of how we have been treated.”* (Community Health Worker FGD ) |
| 5 | *“Personally, …… I knew …all [Volunteer Health Worker] and I organized several sessions to train them in all their operational areas assisted by the CHO, the district environmental health officer and some others”* (DDHS-1) |
| 6 | *“…… when you look at the CHPS’ concepts or the CHPS package for volunteers, it’s the mandate of the community health committee to supervise the activities of the volunteers and the CHO. So it becomes the responsibility of the community to see to the welfare of the volunteers. With welfare, I mean motivation and other petty things. So when we are not able to do it and then community members are also not able to do it, that’s where the challenge lies. As he is saying, the spirit is dying. If they are not paid, the community is supposed to motivate them but the community is also not doing their best to motivate them so that is the challenge.”* (DDHS-2) |
| 7 | *“It was a regional policy by the then Regional Director, the late Dr Nyonator to send all of his DHMTs there [Navrongo CHPS experiment] …. We were there as [A study district] Team…Myself, my public health nurse and disease control officer. We were four.”* (DDHS-1)  *“The trip to the Upper East actually opened (…) our eyes to things that we were supposed to do and we are not doing because when we went to the CHPS compound, the CHOs took us through how they work in the CHPS compound. At first, we were not asking (…) we don’t have the maps stating where this one is, we didn’t have it here (…) and the facilitative monitor visit that they are doing, supervision that they are doing, the guide they are using, is so simple. You have that for the …. Sub-district teams. So that also helped us to form a sub-district team to be doing their own monitoring and supervision. They drew the chart which we didn’t know; we didn’t know it before we went. The community participation was there; I mean their commitment, we saw it.”* (DDHS-2) |
| 9 | *“The volunteers were so important before we could regard the zones as completed. Without them no completed zone.”* (DDHS-1) |
| 10 | *“We approached the Chief of the village with the idea and he was very receptive. Then we met the community leadership and the entire community at a durbar and explained the CHPS concept for their acceptance.”* (DDHS-1) |
| 11 | *“Yes, to get the community members involved right from the first step. For all the 15 steps that we are supposed to cover, at every [first] step we need to involve the community members. This is what we have not done effectively in the past and that is why every structure is destroyed and community members are finding it difficult to renovate them. But I feel from the beginning, before we sent the CHOs to engage the place, if we were to engage the community members we wouldn’t be having these challenges now.”* (DDHS-2) |
|  | *“The Chief identified an accommodation unit which we used as the CHPS compound. We solicited the support of the district political authorities, and began mobilizing resources like equipment, medicines, motor bikes and the like to prepare for CHPS launching.”* (DDHS-1) |
| 12 | *“To support what he said, you know when you look at the CHPS’ concepts or the CHPS package for volunteers, it’s the mandate of the community health committee to supervise the activities of the volunteers and the CHO. So it becomes the responsibility of the community to see to the welfare of the volunteers. With welfare, I mean motivation and other petty things. So when we are not able to do it and then community members are also not able to do it, that’s where the challenge lies.”* (DDHS-2) |
| 13 | *“We involved them in other mass activities. They were well regarded by the Chief and the committee members. And they were always seen with madam [referring to the nurse], all of which fed into their feeling of importance about what they were doing”* (DDHS-1) |
| 14 | *“We are the people working on the ground for the directors work to be easier but here is the case where he does not even know us and to talk of appreciation. This place was the first community that the CHPS was built so many people from other villages come to do a lot of workshop and training for the workers.”* (Community Health Volunteers FGD) |
| 15 | *“Last time when we went for the regional CHPS workshop… I think that was 2015. All the functional CHPS compounds I am not sure they were up to 6. So it’s making things difficult in the community engagements. If you don’t make them know that this is the part they are supposed to play in implementing CHPS in the community, it is very difficult even going to the assembly man telling him that our facility is very bushy and that we need the community to help us to clear the weeds. The assembly man can call the people but you will see that it is just a hand full of people who will come around. This person comes to weed a portion and says I’m done and he’s gone then the other person comes to weed another portion then you realize that the work is not done well.”* (Community Health Volunteers FGD) |
| 16 | *“Yes, we got bicycles from the Ministry of Health/Ghana Health Service. The medications and the supplies they used were supplied by the CHO who in turn sourced them from the District Office….”* (DDHS-1) |
| 17 | *“So some of them are moving out [discontinuing] and some also still do the work. So the logistics aspects concerning the [health information management] registers, they have… but ideally they are supposed to get “wellington boots” and raincoats, torch lights and those things but those ones are no more coming in; rain coats and other things, for some time now we have not been providing for them apart from the registers.”* (DDHS-2) |
| 18 | *“They used to hold stock of condoms, Oral Rehydration Salt (ORS) for diarrhoea cases and some essential medications as first responders. They delivered training services according to their training at the household level.”* (DDHS-1) |
| 19 | *“… you know these volunteers they are not supposed to be paid. So we give the community based surveillance volunteers registers and they are not supposed to be paid so we give the registers with signs and conditions [restrictions]….. It’s the mandate of the community health committee to supervise the activities of the volunteers and the CHO. So it becomes the responsibility of the community to see to the welfare of the volunteers….”* (DDHS-2) |
| 20 | *“…to get the community members involved right from the first step. For all the 15 steps that we are supposed to cover, at every step we need to involve the community members. This is what we have not done effectively in the past and that is why every structure is destroyed and community members are finding it difficult to renovate them. But I feel from the beginning, before we sent the CHOs to engage the place, if we were to engage the community members we wouldn’t be having these challenges now.”* (DDHS-2) |
| 21 | *“It was a regional policy by the then regional director, the late Dr. Nyonator to send all his DHMTs there [Navrongo, Upper East Region]…..”* (DDHS-1) |
| 22 | *“Personally, because this was my first experience, I knew them all [Volunteers] and I had organized several sessions to train them in all their operational areas assisted by the CHO, the district environmental health officer and some others…”* (DDHS-1) |
| 23 | *“Upper East actually (…) opened our eyes to things that we were supposed to do and we are not doing because when we went to the CHPS compound, the CHOs took us through how they work in the CHPS compound. At first we were not asking (…) we don’t have the maps stating where this one is, we didn’t have it here and then we (…) how (…) they wake up (…) because when we went, they had a detail map telling us that, the people in the community wake up at this time, they do this, they do that till the end of the day…..”* (DDHS-2) |
| 24 | *“I think one of the key things that we learnt is strengthening our sub-district teams. So, we started something as a district even before we went to the Upper East region and when we came back, we realized one of the key things that led to their success story was sub-district teams that weree well strengthened to support the CHPS zones. They mentor the CHPS zones, so they are in constant communications with the CHPS zones and then how services are being delivered, and supporting and supervising their activities; because the CHPS zones are many*…..” (CHPS Coordinator) |
| 25 | *“You know, one other area that maybe if it were working, volunteer motivation will also work is when we have the community health management committee working. So you know, these community health management committees have also been trained… like how we train the CHOs, they also have a structured training that they are given. So when they also understand their roles well as a committee, they can easily mobilize the community to know the importance of these volunteers…”* (CHPS Coordinator) |
| 26 | *“It has … it helped me to learn more about how to engage the community and get the community to be involved in whatever we are doing at the CHPS level*…..” (DDHS-2) |
| 27 | *“So they do the training and then we have about hundreds of our volunteers, community health volunteers who have been reoriented by the CHPS Plus program. And then we have the CHPS Plus program also supporting us to equip our volunteers.”* (DDHS-2) |
| 28 | *“So, the sub-district concept is working in [locality named] well now because we have the structures, we have the teams and then they do their own thing, we are using tools, monitory and supervision tools. We got it from Upper East, so we gave them and are using them to do the monitoring.”* (DDHS-2) |
| 29 | *“…we started, though currently out of our fifteen zones about five have structured community emergency response systems. They all have a means of transporting the person to the next level from a CHPS compound to the sub-district level then to the hospital which has been supported by the CHPS Plus program. This therefore means the community is also playing some role by supporting with some token in maintaining the facility.”* (CHPS Coordinator) |
